# Procedure-related vasospasm and reperfusion vulnerability during mechanical thrombectomy

**DOI:** 10.3389/fneur.2026.1842242

**Published:** 2026-06-16

**Authors:** Tarek El Halabi, Rim Araoui

**Affiliations:** Department of Neurology, American University of Beirut Medical Center, Beirut, Lebanon

**Keywords:** mechanical thrombectomy, microvascular dysfunction, milrinone, no-reflow, phosphodiesterase-3 inhibition, platelet activation, vasospasm

## Abstract

Mechanical thrombectomy (MT) has reshaped the treatment of acute ischemic stroke caused by large-vessel occlusion (LVO), yet successful angiographic reperfusion does not reliably translate into meaningful neurological recovery. Landmark randomized trials and the Highly Effective Reperfusion Evaluated in Multiple Endovascular Stroke Trials (HERMES) patient-level meta-analysis established MT as standard of care, but they also brought into focus the persistent problem of incomplete tissue-level rescue despite technically successful recanalization. Procedure-related vasospasm is usually handled as a transient intraprocedural complication, commonly with intra-arterial calcium-channel blockers, and is rarely considered within the broader biology of reperfusion failure. In this mini-review, we propose that vasospasm during endovascular thrombectomy (EVT) should be reinterpreted as a visible marker of a more complex reperfusion-failure state. This state may encompass endothelial injury, distal embolization, inflammatory leukocyte–endothelial interactions,platelet-rich microthrombi, pericyte-mediated capillary constriction, and microvascular no-reflow. Within this framework, intra-arterial vasodilators warrant renewed attention not only for their ability to restore conduit vessel diameter, but also for their potential to influence the quality of tissue perfusion. This perspective is particularly relevant for phosphodiesterase-3 (PDE3) inhibitors such as milrinone, whose pharmacological profile combines vasodilatory effects with biologically demonstrable modulation of platelet activity. Beyond conduit vessel relaxation, such agents may act at the interface between macrovascular recanalization and microvascular reperfusion. We propose that procedure-related vasospasm may identify a subgroup of patients in whom successful recanalization fails to secure effective microvascular reperfusion, and that future studies should evaluate vasodilator strategies using endpoints that extend beyond angiographic resolution to include tissue perfusion, infarct evolution, and functional outcome.

## Introduction

Three pivotal 2015 thrombectomy trials including Endovascular Treatment for Small Core and Anterior Circulation Proximal Occlusion With Emphasis on Minimizing CT to Recanalization Times (ESCAPE), SWIFT PRIME, and EXTEND-IA, along with the subsequent Highly Effective Reperfusion Evaluated in Multiple Endovascular Stroke Trials (HERMES) collaboration established endovascular thrombectomy (EVT) as an effective treatment for anterior-circulation large vessel occlusion (LVO) stroke (1–4). At the same time, the field adopted reperfusion grades such as modified Thrombolysis in Cerebral Infarction (mTICI) as the procedural benchmark for success (5). However, subsequent clinical and imaging studies demonstrated that successful angiographic reperfusion does not necessarily translate into effective tissue salvage or favorable neurological recovery. This recognition gave rise to related concepts including futile recanalization, clinically ineffective reperfusion, and tissue-level no-reflow, in which macrovascular reopening may coexist with persistent downstream hypoperfusion (6–10). That mismatch is commonly discussed in relation to distal embolization, repeated device manipulation, and persistent microvascular dysfunction despite successful recanalization (6–12). Procedure-related vasospasm, despite being familiar to operators, has received substantially less conceptual attention. Reviews of EVT complications consistently include vasospasm among device-related adverse events, yet it is often framed as transient, manageable, and clinically secondary to the main event of clot retrieval (13–15). This framing may be too narrow. The growing vasospasm literature suggests that vasospasm during EVT is associated with technically demanding procedures, repeated device manipulation, and less favorable procedural or imaging outcomes, although direct evidence linking angiographic vasospasm to microvascular no-reflow remains limited and unproven (15–20).

**Figure 1:**
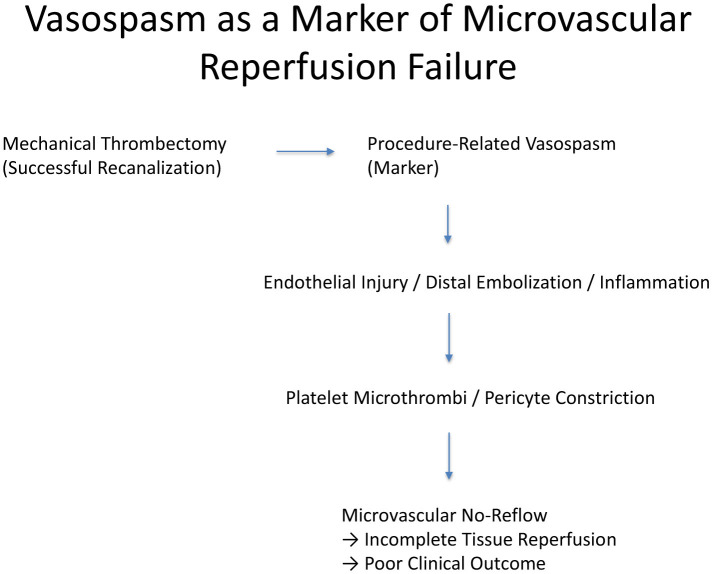
Conceptual framework illustrating procedure-related vasospasm as an angiographic marker of reperfusion vulnerability during mechanical thrombectomy. Repeated device manipulation and vessel-wall irritation during technically demanding EVT procedures may coexist with endothelial injury, distal embolization, inflammatory activation, and vasospasm. These interrelated processes may contribute to downstream microvascular dysfunction, incomplete tissue reperfusion, and unfavorable clinical outcomes. The figure is intended to reflect an associative rather than a strictly causal relationship between angiographic vasospasm and microvascular no-reflow.

Accordingly, the central thesis of this review is not that procedure-related vasospasm represents definitive evidence of microvascular no-reflow, but rather that it may identify a subgroup of thrombectomy procedures characterized by greater reperfusion vulnerability. A conceptual overview of this proposed framework is illustrated in Figure 1. Under this interpretation, angiographic vasospasm is viewed as a potential intraprocedural sign occurring during technically demanding procedures characterized by repeated device manipulation, vessel-wall irritation, endothelial perturbation, and other processes associated with reperfusion vulnerability. These processes have independently been linked to incomplete tissue-level reperfusion, although a direct mechanistic relationship between angiographic vasospasm and capillary no-reflow remains unproven. Accordingly, intra-arterial vasodilators should not be interpreted as validated therapies for no-reflow, but rather as adjunctive agents whose effects on vascular tone, platelet activity, or microcirculatory physiology may warrant further investigation. At present, no clinical or imaging biomarker study has validated angiographic vasospasm as a surrogate marker of microvascular no-reflow during EVT.

## Procedure-related vasospasm during EVT: frequency, predictors, and clinical signal

Procedure-related vasospasm is well recognized during thrombectomy, especially in younger patients, distal or medium-vessel manipulations, and procedures requiring repeated passes or more aggressive stent-retriever interaction with the vessel wall (12, 15, 17, 18). Reported rates of procedure-related vasospasm during EVT vary across studies depending on patient selection, thrombectomy technique, and definitions used, but available series generally suggest frequencies ranging from approximately 3 to 10% (15–19). Although not among the most common EVT complications, vasospasm remains clinically relevant because it frequently occurs in technically demanding procedures involving repeated vessel manipulation or multiple thrombectomy passes. In a single-center analysis, Jesser et al. (15) identified younger age, lower pre-stroke disability, and a greater number of thrombectomy maneuvers and stent-retriever passes as predictors of vasospasm during anterior-circulation EVT. Survey data further suggest that neurointerventionalists remain divided about the clinical importance of vasospasm, which itself is telling: practice heterogeneity often reflects an evidence gap rather than a trivial phenomenon (17).

Clinical outcome data are increasingly difficult to dismiss. In a retrospective single-center cohort study by Jesser et al. (16) intra-arterial nimodipine efficiently resolved angiographic vasospasm, yet patients with vasospasm had greater infarct growth and worse follow-up ASPECTS, indicating that angiographic improvement may underestimate downstream injury. A later prophylactic strategy—adding nimodipine to the guide-catheter flush—was associated with a lower rate of vasospasm without obvious clinical harm, implying that vasospasm is at least partially modifiable (18). Most importantly, in the multicenter Endovascular Treatment in Ischemic Stroke (ETIS) registry, Ferhat et al. (19) studied 13,678 EVT-treated patients and found that vasospasm requiring intra-arterial nimodipine was associated with lower odds of excellent outcome and lower odds of perfect mTICI3 reperfusion, with the strongest negative signal seen for intracranial vasospasm.

These observations align with broader procedural data. Device-related complication studies and complication reviews have long noted vasospasm alongside dissection, perforation, and embolization to new territory (13–15). While a causal chain cannot be established from retrospective analyses alone, the recurring association between vasospasm, multiple passes, and worse surrogate or clinical outcomes suggests that vasospasm may be associated with procedural stress and reperfusion vulnerability (12–15, 19). Importantly, this association should not be interpreted as evidence of causality. Vasospasm may instead represent a non-specific marker of procedural complexity, repeated device manipulation, distal navigation, or vessel-wall irritation during technically challenging thrombectomy procedures.

## From angiographic vasospasm to tissue-level no-reflow

An important reason vasospasm may warrant further attention is that stroke treatment success is not determined solely by reopening the proximal artery. Ter Schiphorst et al. (6) demonstrated tissue no-reflow despite complete recanalization after thrombectomy, directly linking full macrovascular reopening with persistent microvascular hypoperfusion on post-treatment imaging. This clinical observation fits the broader concept of clinically ineffective reperfusion, comprehensively reviewed by Nie et al. (7) in which successful endovascular reopening fails to deliver functional benefit because tissue-level reperfusion remains incomplete or unstable.

The modern stroke no-reflow literature provides a mechanistic scaffold for this interpretation. Sperring et al. (8) synthesized the experimental and translational evidence for no-reflow after stroke recanalization, emphasizing endothelial swelling, microthrombi, leukocyte plugging, pericyte constriction, loss of vasomotor regulation, and blood–brain barrier injury as converging mechanisms. Mujanovic et al. (9) showed in their systematic review and meta-analysis that no-reflow is not merely a preclinical curiosity but a clinically detectable phenomenon associated with worse outcomes after reperfusion therapy. Complementary contemporary reviews by Jia et al. (10) further underscore that microvascular obstruction may persist after both thrombolysis and thrombectomy, and that the field still lacks standardized definitions, measurements, and treatment algorithms.

In that setting, procedure-related vasospasm should not be considered synonymous with microvascular no-reflow. Reduced distal perfusion in the presence of vasospasm may be explained directly by upstream arterial narrowing without necessarily implying capillary-level dysfunction. Nevertheless, vasospasm may coexist with broader reperfusion abnormalities in some procedures characterized by endothelial injury, distal embolization, inflammatory activation, or impaired microvascular reopening. Severe vasospasm may transiently impair distal flow dynamics and complicate further device navigation, although whether angiographic vasospasm directly contributes to capillary-level no-reflow remains uncertain. More importantly, vasospasm may represent an angiographic manifestation of broader procedural and endothelial perturbation occurring during technically challenging thrombectomy procedures (11, 19, 20). Once this broader framework is adopted, the key question shifts from “did the narrowed artery relax?” to “did tissue perfusion normalize?” (6–10, 21).

## Biological plausibility: endothelial injury, inflammation, distal embolization, and capillary failure

Mechanical injury to the arterial wall is biologically plausible and increasingly documented. Abraham et al. (20) used high-resolution contrast-enhanced vessel-wall MRI after stent-retriever thrombectomy and showed signal enhancement consistent with vessel-wall injury in treated segments. Distal embolization is another relevant mechanism: Chueh et al. (11) demonstrated *in vitro* that both stent-retriever thrombectomy and aspiration strategies can generate distal embolic fragments, potentially contributing to downstream microvascular obstruction. This is further supported by Renú et al. (22) who showed that adjunct intra-arterial alteplase after successful thrombectomy (mTICI 2b/3) improves 90-day outcomes, likely by reducing no-reflow due to distal microvascular obstruction. Repeated passes further worsen the biological and clinical picture; Baek et al. (12) reported that five or more stent-retriever passes become futile in terms of successful recanalization and functional outcome, reinforcing the idea that escalating mechanical manipulation may shift the procedure from rescue to injury.

No-reflow biology adds older but foundational microvascular evidence. Mori et al. (21) showed in a primate model that inhibition of polymorphonuclear leukocyte adherence suppresses no-reflow after focal cerebral ischemia, directly implicating leukocyte–endothelium interactions in microvascular obstruction. Okada et al. (23) then demonstrated reperfusion-associated expression of P-selectin and intercellular adhesion molecule-1 after focal brain ischemia, providing a mechanistic link between endothelial activation and leukocyte-mediated capillary plugging. More recently, Shrouder et al. (24) used longitudinal two-photon imaging to show that persistent pericyte dysfunction contributes directly to capillary no-reflow after stroke, strengthening the concept that capillary constriction is not merely theoretical but structurally mediated and sustained.

Taken together, these data support a unified model in which EVT can succeed at the level of clot extraction yet leave important components of reperfusion biology unresolved: vessel-wall trauma may provoke vasoconstriction; embolic debris may fragment distally; activated endothelium may recruit leukocytes and platelets; and pericytes may continue to constrict capillaries despite proximal reopening (6–12, 20, 21, 23–25). Under this model, vasospasm is clinically important not because every narrowed segment directly causes infarct growth, but because it may occur in procedures characterized by greater reperfusion vulnerability and tissue-level perfusion instability.

## Intra-arterial vasodilators at the macro–microvascular interface

In current practice, the most familiar intra-arterial vasodilators for procedure-related vasospasm are calcium-channel blockers such as nimodipine, nicardipine, and verapamil. The contemporary EVT literature around vasospasm is dominated by nimodipine, both as targeted rescue therapy and as prophylaxis via catheter flush (16–19). These agents are attractive because they can visibly relax spastic conduit vessels and restore luminal diameter quickly. That practical efficacy explains why many operators use them despite the absence of standardized protocols (16–19).

However, conduit-vessel spasm relief should not be mistaken for proof of restored microvascular perfusion. The Jesser et al. (16) and Ferhat et al. (19) studies are particularly instructive here: nimodipine-treated vasospasm often resolved angiographically, yet the associated patients still had signals of worse infarct evolution or worse outcome. This does not establish harm from nimodipine; rather, it suggests that angiographic vasodilation alone may be an insufficient endpoint. If vasospasm is nested within a broader syndrome of endothelial injury and no-reflow, then a vasodilator that acts mainly on proximal smooth muscle may improve the picture without fully correcting the biology (6–10, 16, 19, 21).

That conceptual limitation is precisely why vasodilator research in EVT should be reframed. Instead of asking which drug best abolishes visible spasm, future studies should ask which agent most effectively restores reperfusion quality—measured by tissue-level perfusion, infarct growth, hemorrhagic transformation, and neurological recovery. This is also where vasodilators with additional antiplatelet or microvascular effects become especially intriguing (6–10, 21, 26–31).

## Milrinone, PDE3 inhibition, and the antiplatelet dimension

Milrinone is pharmacologically distinct from calcium-channel blockers. As a phosphodiesterase-3 (PDE3) inhibitor, it increases intracellular cyclic AMP, producing vasodilation while also exerting demonstrable effects on platelet activation and aggregation (26, 27). Barradas et al. (26) showed that milrinone inhibits human platelet shape change, aggregation, and thromboxane A2 synthesis *in vitro*, while Horn et al. (27) demonstrated that PDE3 inhibition modulates platelet–monocyte aggregate formation, highlighting its interaction with platelet-inflammatory pathways.

This dual mechanism is conceptually relevant in the setting of no-reflow, where microvascular obstruction is thought to involve platelet-rich microthrombi in addition to leukocyte plugging and capillary constriction (8–10, 21, 23–25). A vasodilator with secondary platelet-modulating properties could therefore be relevant at two complementary levels: restoration of conduit vessel caliber and attenuation of microvascular platelet activity. Supporting this biologic plausibility, Hase et al. (25) demonstrated that cilostazol, another PDE3 inhibitor, reduced no-reflow and hemorrhagic transformation in experimental focal cerebral ischemia.

In the context of EVT-related vasospasm, the key point is not that milrinone is an established antiplatelet therapy in LVO stroke—it is not. Rather, the point is that milrinone occupies a mechanistically richer space than simple conduit vasodilators. Its biology supports a hypothesis that merits dedicated testing in procedure-related vasospasm precisely because the latter may be an interface syndrome between large-vessel constriction and microvascular obstruction (8–10, 21, 23–27).

Clinical feasibility of milrinone in neurovascular vasospasm is further supported by experience in aneurysmal subarachnoid hemorrhage, where both intra-arterial and systemic administration have been associated with angiographic and clinical improvement (28–31).

## Why milrinone is hypothesis-generating in EVT vasospasm

Milrinone is well described in the subarachnoid hemorrhage vasospasm literature. Early case series by Arakawa et al. and Fraticelli et al. reported angiographic and clinical improvement with intra-arterial plus intravenous milrinone strategies for cerebral vasospasm after aneurysmal subarachnoid hemorrhage (28, 31). Lannes et al. (29) later operationalized the Montreal Neurological Hospital protocol, and Lakhal et al. (30) provided stronger observational evidence through the MILRISPASM controlled before–after study. These studies belong to a different disease state, and they cannot simply be imported into EVT-treated ischemic stroke. Nevertheless, they demonstrate that milrinone is feasible in neurovascular vasospasm and that its cerebrovascular effects are not merely theoretical (28–31).

The translational relevance therefore remains exploratory: procedure-related vasospasm during thrombectomy may represent one setting in which milrinone's combined vasodilatory and platelet-modulating effects could warrant further investigation. In this context, milrinone could be studied not as a substitute for thrombectomy, and not as a conventional antiplatelet strategy, but as a targeted adjunct when a procedure has likely injured the vessel wall and threatened downstream perfusion. A reasonable hypothesis is that milrinone may be especially valuable in intracranial vasospasm after multiple passes, distal embolic suspicion, or incomplete tissue reperfusion despite high-grade mTICI scores (6–8, 11, 12, 19, 20, 26–31).

At the same time, the field should avoid overstatement. The existing stroke EVT literature does not establish milrinone as effective for procedure-related vasospasm, nor does it prove that any antiplatelet effect translates into better capillary reperfusion. Milrinone remains hypothesis-generating because its mechanism intersects with biological pathways implicated in no-reflow, not because outcome evidence in EVT currently exists (8–10, 21, 26–31). Moreover, milrinone administration warrants stringent clinical supervision due to its known association with peripheral vasodilation and systemic hypotension, particularly when administered systemically and at higher dosages (32).

## Research agenda for a mini-review framework

Several concrete research priorities follow from this framework. First, vasospasm should be phenotyped more rigorously by location (intracranial vs. cervical), severity, timing, number of associated passes, and coexistence with distal embolization or vessel-wall injury (11, 12, 15–20). Second, studies should move beyond immediate post-vasodilator angiographic appearance and include tissue-level reperfusion metrics such as perfusion imaging, infarct growth, and hemodynamic assessment after the procedure (6–10, 21). Third, comparative pharmacology should be formalized: nimodipine or other calcium-channel blockers could be compared with milrinone in prespecified EVT vasospasm cohorts, while tracking blood pressure effects, hemorrhagic events, and functional outcomes (16–19, 26–31).

Finally, trial design should reflect the underlying biology. A future study of vasodilators in EVT should not simply enroll all-comers with any angiographic narrowing. It should target patients in whom vasospasm is most likely to mark reperfusion failure—such as intracranial spasm, multiple device passes, mTICI–perfusion mismatch, or early imaging evidence of no-reflow (6–10, 12, 19, 21). This framework may help future studies better define patient selection, endpoint choice, and adjunctive vasodilator strategies in EVT-related vasospasm.

## Conclusion

Procedure-related vasospasm during thrombectomy may represent an angiographic sign of procedures characterized by greater reperfusion vulnerability, including vessel-wall injury, distal embolic burden, endothelial activation, and microvascular dysfunction (6–12, 15–21, 23–25).

Within that framework, intra-arterial vasodilators may warrant investigation not only for angiographic spasm resolution but also for their potential influence on tissue-level reperfusion. Calcium-channel blockers remain the best-described agents in current EVT practice, but milrinone is especially compelling as a hypothesis-generating adjunct because PDE3 inhibition offers a biologically plausible bridge between vasodilation and platelet modulation (16–19, 25–31). Although a direct mechanistic relationship between angiographic vasospasm and capillary no-reflow remains unproven, the observed association between vasospasm, procedural complexity, and unfavorable outcomes warrants further investigation. Future studies should evaluate whether recognizing vasospasm as a marker of reperfusion vulnerability can improve patient selection, endpoint choice, and adjunctive therapy design in EVT.
